# Hierarchical Heteroaggregation of Binary Metal-Organic Gels with Tunable Porosity and Mixed Valence Metal Sites for Removal of Dyes in Water

**DOI:** 10.1038/srep10556

**Published:** 2015-05-27

**Authors:** Asif Mahmood, Wei Xia, Nasir Mahmood, Qingfei Wang, Ruqiang Zou

**Affiliations:** 1 Department of Materials Science and Engineering College of Engineering, Peking University, Beijing 100871 (P. R. China)

## Abstract

Hierarchical heteronuclear metal-organic gels (MOGs) based on iron (Fe) and aluminium (Al) metal-organic framework (MOF) backbones bridged by tri-carboxylate ligands have firstly been synthesized by simple solvothermal method. Monometallic MOGs based on Fe or Al give homogenous monoliths, which have been tuned by introduction of heterogeneity in the system (mismatched growth). The developed gels demonstrate that surface areas, pore volumes and pore sizes can be readily tuned by optimizing heterogeneity. The work also elaborates effect of heterogeneity on size of MOG particles which increase substantially with increasing heterogeneity as well as obtaining mixed valence sites in the gels. High surface areas (1861 m^2^/g) and pore volumes (9.737 cc/g) were obtained for heterogeneous gels (0.5Fe-0.5Al). The large uptakes of dye molecules (290 mg/g rhodamine B and 265 mg/g methyl orange) with fast sorption kinetics in both neutral and acidic mediums show good stability and accessibility of MOG channels (micro and meso-/macropores), further demonstrating their potential applications in catalysis and sorption of large molecules.

The coordination chemistry has risen to another step with introduction of porous coordination polymers, more commonly known as metal-organic frameworks (MOF), which offer high surface areas, flexibility over controlling their structure, tunable pore volume and pore size distribution^1^,^2^,^3^,^4^,^5^,^6^,^7^,^8^. Precise control over MOF structure has triggered wide range of applications of these giant molecules in the fields of catalysis, gas sorption, drug delivery and energy storage^9^,^10^,^11^,^12^,^13^. The possibilities of tuning the ligands with various functional groups or varying their lengths to adjust the resulting properties provide a unique feature of material fabrication. MOFs can be tailored by several methods including mechanochemical, electrochemical, solvothermal and microwave assisted growth^14^. Simple synthesis by these methods lead to entangled structures which have been addressed by use of different solvents, structure directing agents, various types of ligands as well as multi-metal systems^5^,^15^,^16^,^17^,^18^,^19^,^20^,^21^,^22^,^23^,^24^. However, MOFs have been synthesized by different methodologies, but the synthesis of meso-/macroporous MOFs while maintaining large surface area remains a big challenge^16^.

On other hand, aerogels have been synthesized by replacement of solvent molecules under supercritical conditions and possess unique characteristics of large empty spaces (extremely low density with up to ~95% volume of air) due to 3-dimensional (3D) entangled networks^25^,^26^. Such gels retain their original structure which is prone to shrinkage if dried under ambient conditions. Among these aerogels, metal-organic gels (MOGs) have been synthesized recently by combining organic and inorganic moieties which take benefits from MOF backbone and keep its inherent structure when dried in the reduced atmosphere^7^,^27^,^28^,^29^,^30^,^31^. MOGs are hierarchical porous metal organic monoliths which exhibit high surface areas, tunable porosities, inherent structure retention, low densities, inherently present open metal sites and large channels for mass transfer, sorption and catalysis^27^,^32^. The development of MOGs has seen several advances recently but rational design still remains a big challenge^33^. Generally, the gelation mechanism involves three steps including i) metal-ligand coordination to form MOF chains, ii) aggregation of MOF chains to conform into MOF particles (MOFP), iii) aggregation of MOFP to construct hierarchical MOG structures. Since final structure of MOG depend on aggregated MOFP, hence, the size of MOFP becomes very important. Several factors such as extensive crystallization or precipitation could affect the MOG inherent structure adversely^26^,^33^,^34^. So, in designing MOG synthesis from MOFP, the most important factor which decides MOG scaffold structure depends on synthesis conditions where gelation process could dominate over crystallization and precipitation. Several methods have been explored to suppress crystallization in the MOG synthesis including use of different solvents, surfactants, inhibiting agents and variations in reaction temperature, pH and time^16^,^35^,^36^,^37^. Furthermore, very recently, the mismatched growth of MOFP (where coordination can be perturbed to control gelation) has been identified as a critical factor in optimizing MOG synthesis^16^. However, despite these advancements, it is very important to explore gelation when different coordination sites are present in the system (multiple metals, which have the ability to coordinate with the same ligand)^20^,^33^,^38^. The heterogeneity in the MOG system can be further enhanced using mixture of metal sources with reactivities at different temperatures toward organic ligand which will disrupt the crystal growth, enhancing gelation over crystallization^15^.

Among MOFs, MIL-100 (*MIL=Materials Institute Lavoisier*) exhibit large surface areas, giant pores^39^ and has been investigated for its structural,^39^,^40^ catalytic and sorption (gas,^21^ water^41^ and other toxins^30^) properties. Monometallic MOGs based on MIL-100 (Fe or Al) have been studied recently for their structural properties and applications. Considering above advantages, here we choose Fe and Al with benzene tri-carboxylate (BTC, as coordinating ligand) to establish unique bimetallic MOG system. These MOGs have been synthesized by simple solvothermal method followed by supercritical drying. These gels can be used to capture macromolecules due to their large pore diameters and pore volumes as demonstrated by appreciable dye uptake in acidic environments where most of the MOFs/MOGs tend to decompose. An easy and scalable synthesis method and fast uptake of large amount of dyes suggest wide range of potential applications of these materials in sorption and catalysis.

## Results

**Figure 1:**
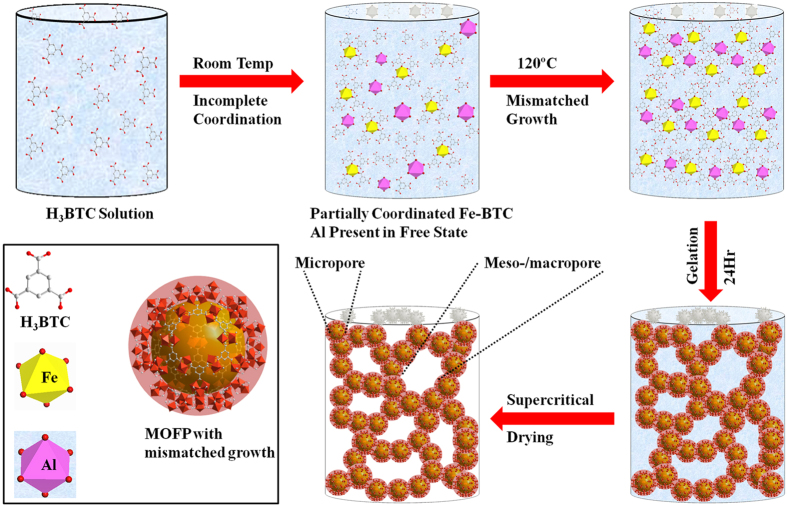
Mismatched growth of mixed metal MOGs.

Several monometallic and bimetallic MOAs and MOXs have been synthesized with different concentrations of metals such as Fe-BTC (MOA-1 and MOX-1), 0.75Fe-0.25Al-BTC (MOA-2 and MOX-2), 0.5Fe-0.5Al-BTC (MOA-3 and MOX-3), 0.25Fe-0.75Al-BTC (MOA-4 and MOX-4) and Al-BTC (MOA-5 and MOX-5), respectively. The growth of MOGs is a coordination driven process which is affected by crystallization or precipitation. The most suitable approach to suppress crystallization is to control the growth of MOF chains. Monometallic system (Fe or Al) leads to homogenous monoliths where growth of MOF chains is only perturbed upon decrease in reactant concentrations which results in increased crytallinity in the product. Here, in this work, the growth process of the MOGs has been addressed by introducing heterogeneity to enhance mismatched growth. Based on reactivity, Fe can coordinate at room temperature with BTC while Al coordinates at considerably higher temperatures (~120 °C) (Figure S1-2 show incomplete gelation in samples with higher concentration of Al)^42^. Therefore, by using this concept, Al was introduced to Fe system to disturb the growth of Fe-based monoliths to obtain gels with heterogeneous MOFP. The growth of MOFP can be divided in two steps for heterogeneous MOGs containing multiple coordinating centers as shown in Fig. 1. In early stage, Fe coordinated with BTC to form small MOF clusters which grow until all the Fe entities were coordinated. The growth of crystallographic chains is perturbed by fast reduction of Fe centers which lead to non-crystallographic supramolecular gels^34^,^36^. Upon increase in reaction temperature, Al coordinate with leftover BTC giving rise to heterogeneous gelation. Figure S3 represent the TEM (transmission electron microscope) analysis and elemental mapping of the gel. It is quite clear that small Fe networks are formed as highlighted while Al is randomly distributed all over the gel^32^,^40^. This further supports our presented scheme to effectively control the crystallization process.

**Figure 2:**
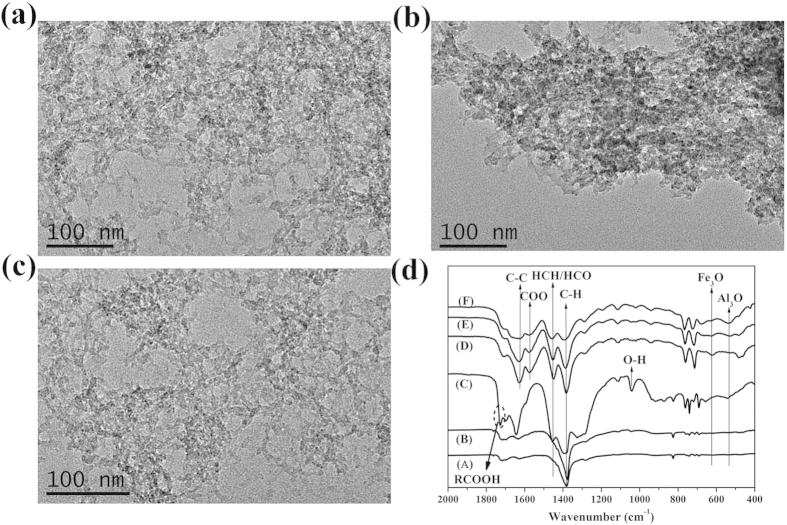
TEM images of (**a**) MOA-2, (**b**) MOA-3, (**c**) MOA-4. (**d**) show FTIR spectra of MOGs synthesized at room temperature [(A) MOA-2@RT, (B) MOA-3@RT, (C) MOA-4@RT] and at 120 °C [(D) MOA-2), (E) MOA-3, and (F) MOA-4].

Control over size of MOFP is also very important in defining the characteristics of resulting MOGs (such as surface area, pore volumes, sorption etc) as the MOFP join together through weak Van der Walls force, H-bonding or π-π stacking^16^. In our designed systems, increasing heterogeneity led to large MOFP in the MOGs. Fig. 2(a-c) represent morphological aspects of MOGs studied using TEM. It is quite clear that size of MOFPs increase as function of increasing heterogeneity. MOA-3 contains equal concentrations of metal salts and results in large MOFPs with average size of 20 nm. But the MOGs (MOA-2 and MOA-4) with higher concentration of a particular metal showed comparatively smaller MOFPs (10-15 nm). For different ratios of the two metals in the reaction chamber, it can be assumed that when Fe^3+^ content is higher in bimetallic MOGs, it can form a network with a small amount of entrapped species (Al^3+^ ions, BTC) generating small networks to give relatively smaller particles. Similarly, when Al^3+^ is in higher concentration, no solid gelation is observed at room temperature leaving small particles of Fe-BTC dispersed in the solution (Figure S1). However, when both metals are equal in concentrations, Fe^3+^ formed large networks with higher amounts of entrapped species (Al^3+^ ions, BTC), which generate secondary network upon heating to provide large MOFP. In addition, heterogeneity is further induced by different ionic sizes of metals like Fe^3+^ (0.067 nm), a transition metal with relatively large size while Al^3+^ (0.055 nm) belong to p-block and has smaller ionic radius. Furthermore, BTC has three possible chelation points (-COOH), thus if both Fe^3+^ and Al^3+^ react with the same BTC molecule, this could also disrupt the crystallization process triggering the gelation process. Hence, incomplete coordination at room temperature due to several factors (reactivity difference, ionic sizes) improved the gelation behavior of the resulting MOGs.

**Figure 3:**
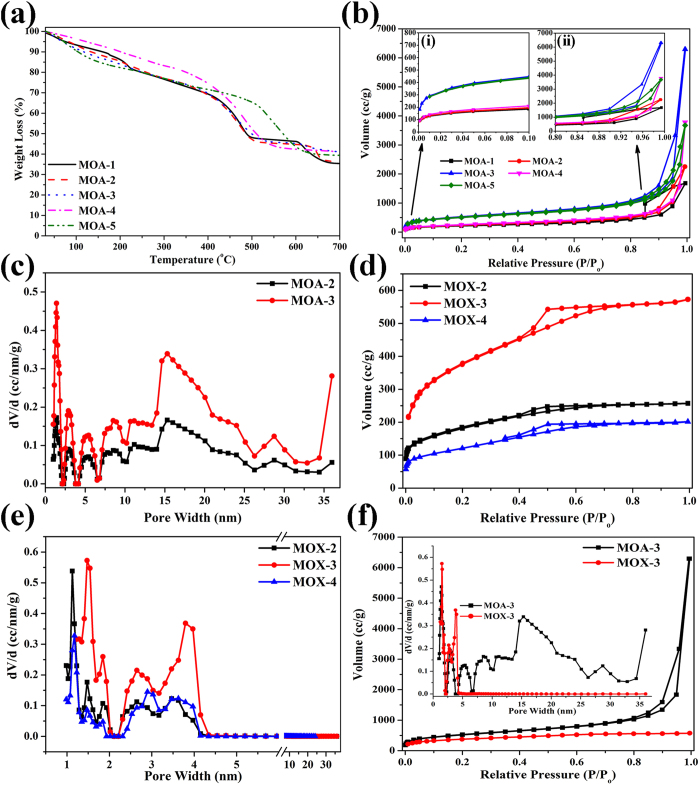
(**a**) Thermal stabilities of MOAs in N_2_ atmosphere, (**b**) N_2_ sorption isotherms and (**c**) pore size distribution of mixed metal MOAs. (**d**) and (**e**) represent N_2_ sorption isotherms and pore size distributions for MOXs respectively. (**f**) Comparison of N_2_ sorption for MOA-3 and MOX-3 and inset shows difference in pore size distribution of respective MOGs.

Temperature variations have been considered another critical parameter to control the properties of resulting MOGs^16^,^35^. To explore the effect of temperature on gelation, mixed metal MOGs were synthesized at different temperatures (room temperature, 80 and 120 °C). TEM analysis exhibited large size MOFPs in gels synthesized at higher temperatures (Figure S4). The Fourier transform infrared spectroscopy (FTIR) spectra of the MOGs synthesized at different temperatures are shown in Fig. 2d. The FTIR spectra of gels synthesized at room temperature show higher amount of unreacted BTC in case of higher Al^3+^ concentration (MOA-4, MOA-5) which further proved our proposed scheme for synthesis of heterogeneous MOGs. The presence of extra absorption band at 1730 cm^−1^ for MOA-4 (Fig. 2d) and MOA-5 (Figure S5a) corresponds to the presence of RCOOH groups which clearly depict the absence of coordination between BTC and Al^3+^^43^. But the presence of very weak band at 1730 cm^−1^ in MOA-2 and MOA-3 (Fig. 2d) synthesized at room temperature suggest small amount of unreacted BTC in the reaction mixture. Since the MIL-100(Fe/Al) consist of Fe_3_O and Al_3_O groups to form the MOF framework, the band at 621 cm^−1^ has been attributed to symmetric stretching vibration of Fe_3_O groups with intensity decreasing upon increasing Al^3+^ concentration (Fig. 2d(D-F))^44^,^45^. Thermal stability of the MOGs was tested in N_2_ atmosphere using thermogravimetric analysis (TGA) and all MOGs show three distinct regions of weight loss as shown in Fig. 3a. The Al-based MOGs show higher stability as compared to Fe-based and mixed metal MOGs owing to difference in electronegativity of the metallic species. The Al-O bond has more ionic character than Fe-O needing higher energy to break. First major weight loss depict degradation of 3D framework and organic species. All the bimetallic MOGs show onset of degradation at similar temperature with minimal differences while MOA-5 show considerable stability up to 550 °C. Above 550 °C, the framework decomposed and formed the Fe_3_O_4_ species (JCPDS No 19-0629) as proved by x-ray diffraction analysis (XRD) of the residue of MOGs heated up to 550 °C and represented in Figure S6a. Further heating led to more weight loss due to conversion of Fe_3_O_4_ into Fe (JCPDS No 65-4899) and iron carbide (JCPDS No. 44-1292), as shown in Figure S6b-c.

The surface areas of the MOGs were calculated using N_2_ sorption at 77 K *via* Brunauer–Emmett–Teller (BET) analysis as shown in Fig. 3b and 3d for MOAs and MOXs, respectively. The microporosity arises from the basic MOF structure and both MOAs and MOXs have similar microporosity due to identical MOFP in the backbone. However, the drying conditions have significant effect on the final structure of these MOGs. To get MOXs, the wet MOGs are dried in air which lead to shrinkage of the porous network due to unprecedented removal of solvent molecules resulting in mesoporosity in MOXs, clearly proved by the hysteresis in the desorption curve. Dried under supercritical conditions, wet MOGs provides MOAs with inherent structure which show high N_2_ sorption isotherm (Type I) at lower pressures clearly showing the microporosity as shown in the inset Fig. 3b(i). Similarly, as higher pressures are approached; large uptakes of N_2_ depict pore condensation which is a property of meso-/macropores showing type IV behavior with significant hysteresis in desorption curves as shown in Fig. 3b(ii). The inset Fig. 3b(ii) show the hysteresis present in the sorption curves at higher pressures. Thus, the MOGs consisted of micro and meso-/macropores which aggregate together hierarchically to form gels and show sorption between type-I and type-IV with dominant sorption behavior towards type-IV isotherms^16^. The rise in adsorption at higher pressures is considerably high for mixed metal MOAs as compared to monometallic MOAs. Similarly the adsorption at higher pressures is very high in MOAs as compared to MOXs with similar concentrations which clearly depict the degradation/shrinkage of large pores in MOXs. Developing meso-/macroporous metal organic complexes has been a critical issue of recent times. MOGs synthesized here by simple solvothermal method exhibit a mixture of micro and meso-/macropores. As shown in Table 1, it is quite evident that pore volume of MOGs increase with increasing heterogeneity in the system. The monometallic MOGs (MOA-1 and MOA-5) exhibit pore sizes dominantly in the range of 10 and 2 nm, respectively with Al-based MOGs also containing some large pores close to 10 nm (Figure S7a-b). The mixed metal MOGs show overall improvement of pore sizes with majority of pores in the meso-/macroporous range as shown in Fig. 3c. A wide pore size distribution in MOA-3 suggests the bridging of metals together through BTC to build large pores. The reactivity difference between the two metals play critical role and lead to higher pore sizes and pore volumes. MOGs with highest heterogeneity (0.5Fe/0.5Al) exhibit high pore sizes (up to 16 nm) and pore volume (9.737 cm^3^/g). Mixed metal MOXs show considerably less uptake of N_2_ in comparison to their MOA counterparts (Fig. 3d) which clearly suggest destruction of channels in MOGs upon drying in air. This fact is further proved by pore size distribution of MOXs shown in Fig. 3e where all the pores are in the range of 1-4 nm which is due to shrinkage of meso-/macropores towards microporous nature. N_2_ sorption isotherm of MOA-3 and MOX-3 show great difference at higher pressures suggesting absence of large pores in xerogels as represented in Fig. 3f. MOX-3 shows a dominating pore size at around 1 and 4 nm and the MOA-3 sample has a quite wide pore size distribution from 1.5 to 16 nm. The collapse of the large pores in MOX-3 sample is further confirmed by the drastic reduction of pore volume (0.88 cm^3^/g). Table 2 represents comparison of the gels synthesized prior to this work. It is worth noting that bimetallic gels provide highest surface areas and high pore volume values along with meso-/macropores.

**Figure 4:**
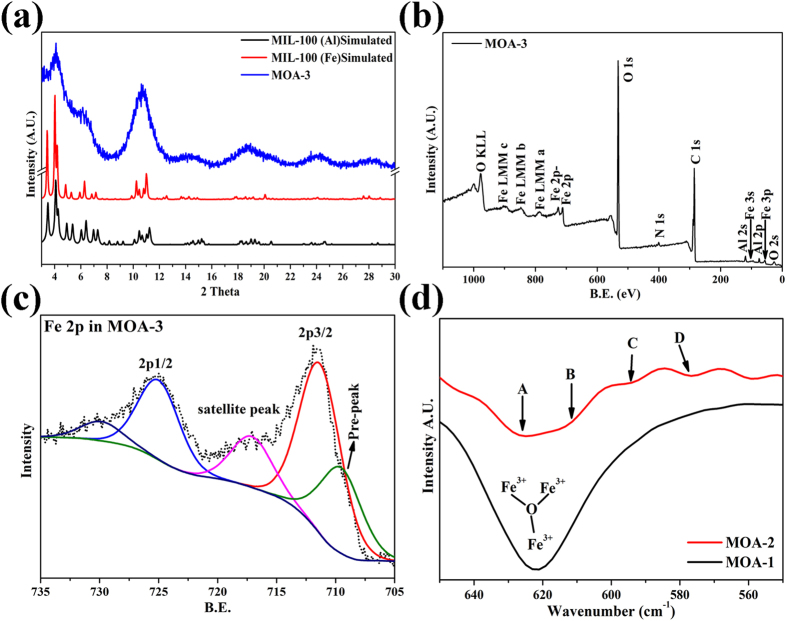
(**a**) XRD patterns of simulated MIL-100 (Fe)^32^ , MIL-100 (Al)^40^ and MOA-3, (**b**) XPS spectrum of MOA-3 and (**c**) fitted XPS spectrum of Fe from MOA-3 and (**d**) FTIR spectra of MOGs.

XRD analysis was used to explore the crystallinity in the MOGs as shown in Fig. 4a and Figure S8. The XRD patterns of the MOGs match with simulated single crystal MIL-100 (Fe/Al). This implies MOG consist of basic MOF structure built by partial chains of Fe- /Al- BTC and their growth is perturbed by incomplete coordination. Considerable broadness in the XRD patterns arises from the nanoparticles which act as building blocks in 3D framework of aerogels and are consistent with the TEM results (Fig. 2a-c) of MOGs^46^. A slight peak shift is also observed from lower to higher angles between 2θ = 10−11° (Figure S8e) from MOA-1 to MOA-5 which shows a transformation in structure upon changing the metallic species in the system^47^. The composition and oxidation states of the elements were further studied by X-ray photoelectron spectroscopy (XPS). Existence of core levels of Fe, Al, C, O and N confirm the presence of all species in the synthesized MOGs as shown in Fig. 4b and Figure S9a,b. The introduction of second metal leads to the reduction of Fe (Fe^3+^ to Fe^2+^), which give mixed valence complexes and provide basis for gelation over crystalline structure^47^,^48^. Fig. 4c represents de-convolution of XPS spectrum for Fe 2p in MOA-3. The doublet observed at 710-712 eV in case of MOA-3 (Fig. 4c) along with pre-peak at 710.3 eV prove the presence of Fe^2+^ with Fe^3+^ species as compared to MOA-1 where single XPS peak is observed at 711.8 eV corresponding to Fe^3+^ (Figure S9c)^49^. However the nature of bonding for both metals is same towards the ligand (Fe_3_O/Al_3_O) but presence of a small XPS peak in MOA-3 for carbon arises due to the change in hybridization of carbon as shown in Figure S10b^50^. The formation of mixed valence gel was further confirmed by FTIR analysis of MOGs. Figure 4d represents FTIR spectra of MOGs between 660-540 cm^−1^. Absorption band at 620 cm^−1^ in case of MOA-1 has been attributed to Fe_3_O group. However, a clear peak shift is observed in case of MOA-2. The regions between A and B in MOA-2 and presence of absorption band at 594 and 576 cm^−1^ prove the presence of mixed valence species with the introduction of Al^45^.

**Figure 5:**
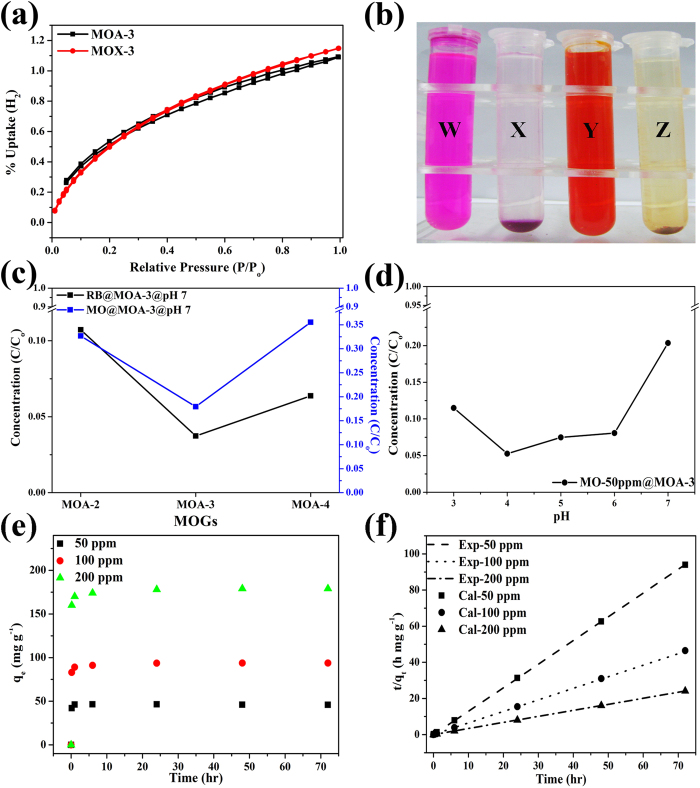
(**a**) H_2_ sorption isotherms of MOA-3 and MOX-3. (**b**) Adsorption of dye polluted water on MOA-3. W and Y represent polluted waters by dyes (RB and MO) and X and Z represent dye adsorbed solutions on MOA-3 for RB and MO, respectively. (**c**) Adsorption of dyes on different mixed metal MOGs at neutral pH (**d**) Adsorption of MO on MOA-3 as function of different pH of solution. (**e**) Adsorption of different concentrations of MO as function of time and (**f**) represent second order kinetics of MO adsorption on MOA-3.

For enhanced sorption, high surface area and pore sizes of aerogels are important parameters as they determine the number of accessible sites and the rate of sorption. Due to presence of mixed metal sites, large surface area, pore sizes and pore volumes, MOA-3 has been further investigated to evaluate the accessibility of its channels for sorption of small and large molecules. Figure 5a represents the hydrogen uptake by MOA-3 and MOX-3 with maximum uptake of 1.09wt% and 1.14wt% of H_2_, respectively. Both MOA-3 and MOX-3 exhibit microporosity (Fig. 3d) and hydrogen can access very small pores (micropores) due to small molecular diameter, so both show almost equal uptake of H_2_. MOX-3 consists of large volume of micropores as compared to MOA-3 which could be leading factor in a slight larger adsorption in MOX-3. The accessibility of pores was further evaluated by large molecule (dye) sorption analysis. Furthermore, the accessibility of pores for dye sorption was also studied in neutral and acidic media. It is always important to develop stable MOG systems which could bear different pH conditions since most MOFs/MOGs decompose in acidic or basic conditions. Here two different dyes are evaluated, methyl orange (MO) and rhodamine B (RB) with diameters of ~1.39 nm and ~1.32 nm, respectively as shown in Figure S11. Figure 5b (W,Y) show pure dye solutions for RB and MO while Fig. 5b (X,Z) represent adsorbed dye solutions onto MOA-3, respectively. At neutral pH, mixed metal MOGs showed higher uptake of dyes with more prominent results for MOA-3 as shown in Fig. 5c and S12a-b. Because of the high surface area and large pore volume, MOA-3 shows the highest uptake of dyes with fast adsorption kinetics at neutral pH. Sorption of dyes on MOGs can be influenced by a number of parameters including pore structure, open metal sites, breathing properties or charge interaction between adsorbent and adsorbate^51^. Thus, pore size and pore volume along with surface area and open metal sites are critical factors in large molecule sorption. MOA-3 possesses wide pore size distribution including micro and meso-/macropores with sizes of 1.4, 2.9, 5.4, 8.5 and 15.31 nm. The presence of large pores comparable to molecular size of dyes provide higher sorption in comparison to other MOGs which have large proportion of micropores smaller than that of size of dye molecules. MOA-3 also contains mixed valence metal sites (Fe^3+^-Fe^2+^) proved by FT-IR and XPS which also influence the adsorption kinetics^24^.

Due to these excellent aforementioned characteristics, MOA-3 has been further studied at differential pH conditions to optimize the conditions for highest uptake. After a series of experiments, it is found that MOA-3 shows highest sorption of dyes at pH ~ 4. Both dyes change into ionic form in the acidic medium which enhanced their adsorption on the metallic sites in the MOGs^44^,^52^. This improved the adsorption kinetics of dyes on the gels. The adsorption kinetics of MO on MOA-3 was evaluated as function of time and concentration and very high initial adsorption capacity of MOG was observed in first 10 minutes which reached equilibrium during 1–6 h (Fig. 5e). Time dependence adsorption kinetics was studied experimentally and pseudo-second-order kinetic model was employed to attain adsorption rate constant against different concentrations. The model description is given in Equation (1).


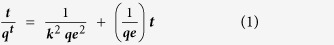


Where *q*_*t*_ and *q*_*e*_ are adsorption capacities (mg/g) at any time *t* (min) and at equilibrium. *K*_*2*_ is the rate constant of pseudo-second order adsorption (g/mg min). The model describes the adsorption capacity of a solid phase by explaining the rate controlling step of chemisorption mechanism. The values obtained by model were in good agreement with experimentally obtained values. The plots of *t/q*_*t*_ against *t* gave a straight line as shown in the Fig. 5f. The *k*_*2*_ and *q*_*e*_ values are given in Table 3. The adsorption capacity at equilibrium (*q*_*e*_) continuously increased as initial concentration of MO increased; whereas adsorption rate constant *k*_*2*_ decreased, respectively that strongly indicate the significance of chemisorption in rate limiting step. The reduction of Fe from Fe^3+^ to Fe^2+^ leave open metal sites in the mixed metal MOGs which have been utilized to adsorb the dyes in large amounts at superior rates by chemisorption at low pH^24^,^51^. The maximum adsorption capacity of MOA-3 was found to be 265 mg/g for MO and 290 mg/g for RB which is much higher than most of the reported MOF and carbon materials for dye sorption^52^,^53^,^54^. These excellent sorption abilities of the developed MOGs highlighted their suitability in the fields of catalysis and sorption.

## Discussions

Development of porous metal organic monoliths has found increased interest recently due to unique properties (surface area, porosity and low density) and wide range of applications^55^,^56^,^57^,^58^,^59^. Despite these progresses, MOG applications have suffered severely due to poor control over their structures, limited understanding of gelation behavior and hurdles in large scale synthesis. Since gelation accompanies crystallization, hence, disruption of crystallization to abort growth of crystalline MOF is very important. The gelation has been primarily studied in monometallic systems with single/multiple ligands. However, very recently, development of mixed metal MOFs has attracted lot of attention due to improved luminescent, magnetic, and sorption properties^21^,^22^,^23^,^24^. Hence, we have demonstrated the growth of mixed metal MOF chains to obtain heterogeneous MOFP which aggregate in 3D network to form hierarchical gels. The gelation process was tailored by employing the relative reactivity of metal centers towards organic ligand. As explained in earlier sections, the MOFP cease to grow after some time due to reduction of reacting species leaving incomplete branches on the surface of the particles which then help in attachment of particles to generate 3D gel (Fig. 1). Furthermore, we have successfully demonstrated that pores in MOGs could be readily tailored using mismatched growth of the MOF chains (Fig. 3). MOGs have been generated from entangled rigid MOF chains which constitute permanent porosities. The MOFP are composed of basic MOF chains entangled together giving rise to microporosity while agglomeration of particles together provide meso-/macroporosity (as shown in Fig. 1). A wider pore size distribution suggest the presence of various coordinating sites giving rise to mismatched growth thus resulting in pores of various sizes. It is found that increasing heterogeneity in the system provide an opportunity to tailor the resulting MOG morphology at micro scale by varying the concentrations of representative inorganic moieties. The pore volumes of the resulting MOGs also increase with increasing heterogeneity as shown in table 1. These heterogeneous gels (MOA-3) exhibit highest pore volumes (9.737 cc/g) reported to date to the best of our knowledge.

Another widely investigated factor for functional application of MOFs is the derivation of mixed valence metallic species with open metal sites in the final product but to the best of our knowledge, open metal sites are not reported before in gels. Monometallic monoliths could only exhibit singular oxidation state without any open metal sites. However, the mixed metal MOGs consisted of open metal sites which expand their potential practical applications in sorption and catalysis. These applications have been elaborated by high uptake of small (H_2_) and large molecules (dye). Although instability of MOFs in aqueous solutions largely limits their applications, as-synthesized MOGs are highly stable in aqueous solutions. Furthermore, most of the industrial effluents are acidic with varying pH conditions which necessitate the stability of adsorbent when used under mild as well as strong pH conditions. MOGs were highly stable in acidic solutions demonstrating potential suitability for catalytic reactions under acidic conditions. Most of textile industrial effluents contain organics (dyes) within the concentrations of 50-100 ppm^60^. MOGs showed high uptake of representative dyes from aqueous solutions both in neutral as well as acidic solutions. Hence, the availability of large empty spaces (high surface area), pore apertures comparable to molecular size of dyes, large pore volume and presence of mixed valence sites contribute to good sorption properties of MOGs. In essence, these developed MOGs show simultaneous control over many characteristic properties such as surface area, gelation, porosity, pore volume and above all provide opportunity to synthesize MOGs at large scale.

In summary, we have demonstrated facile and scalable method which can be generalized to develop aerogels with heterogeneous MOFPs comprising of different metals. The gels consist of basic MOF backbone giving a 3D scaffold which assemble together to give MOFPs eventually joining together upon gelation to form MOGs. The gelation process demonstrated an increase in particle size of MOFP as a function of increasing heterogeneity in the MOG system. As a result, MOGs with largest heterogeneity consist of relatively large particles (~20 nm). In contrast to monometallic counterparts, bimetallic MOGs exhibit high surface areas, pore diameters/sizes and overall pore volumes and highest values of these properties are found with maximum heterogeneity. The MOGs show high uptake of dyes under acidic conditions. MOGs can uptake 290 mg/g of RB and 265 mg/g of MO dye from the solution. The adsorption coefficient was also found in correlation with pseudo second order kinetic model of adsorption. We believe that synthesis of bimetallic gels presented here will be an important step towards development of non-crystalline MOGs with a fine tuning of surface area, pore size and pore volume for various important applications including sorption and catalysis.

## Methods

### Synthesis

MOGs were synthesized by typical reaction between metals (M = Fe and Al) and 1,3,5-benzene tri-carboxylic acid (H_3_BTC) in ethanol as explained in literature^30^. For the synthesis of monometallic MOGs, metal salt (Fe(NO_3_)_3_.9 H_2_O or Al(NO_3_)_3_.9H_2_O) solutions were adjusted to 0.015 mol for each of respective metals while the concentration of H_3_BTC was 0.01 mol. Bimetallic MOGs were synthesized using variable concentrations of metals while total concentration of metal was maintained at 0.015 mol. The metal salt and H_3_BTC were mixed in ethanol separately to make homogenous solutions at room temperature. These solutions were then mixed and transferred to steel container to heat them up to 120 °C for 24 hours. The samples were then subjected to supercritical drying to get aerogels. In other reactions, xerogels were also obtained by drying the samples under air.

### Characterization

MOGs were characterized by powder XRD by using Bruker D8 advanced diffractometer (40 kV, 40 mV) using Cu-Kα radiation (2θ = 3-50 at scan rate of 4 °/min), Thermogravimetric analysis, up to 800 °C with heating rate of 10 °C/min (TA Instruments SDT Q600 Analyzer). The surface area of the MOGs was calculated using N_2_ sorption test in Quantachrome autosorb-IQ gas adsorption analyzer at 77 K. All samples were evacuated at 150 °C for 5 hours under dynamic vacuum before adsorption test. Using sorption data, non linear density functional theory (NL-DFT) was applied to calculate the pore size distribution while pore volumes were calculated at relative pressure (P/P_o_) of 0.995. The transmission data was collected using Fourier Transform infrared spectrometer (Bruker ectro 22) between 4000-400 cm^−1^. XPS was used to find the oxidation states of the metals in the respective samples (Kratos Analytical Ltd). The microstructure of MOGs was studied with Transmission Electron Microscope (TEM, FEI Tecnai T20) and Field-emission High Resolution Transmission Electron Microscope (JEM-2100F).

### Adsorption Experiments

To evaluate the accessibility of MOG channels, large molecules (dye) sorption was studied. Before sorption experiment, the aerogels were evacuated in a vacuum oven overnight. Stock solutions (1000 ppm) of dyes were made with 0.01 M NaCl. In a single sorption experiment, 10 mg (±1 mg) of MOA was taken in a vial with Teflon lined screw cap and 10 ml of dye solution was added to test sorption capability.

## Additional Information

**How to cite this article**: Mahmood, A. *et al.* Hierarchical Heteroaggregation of Binary Metal-Organic Gels with Tunable Porosity and Mixed Valence Metal Sites for Removal of Dyes in Water. *Sci. Rep.* **5**, 10556; doi: 10.1038/srep10556 (2015).

## Supplementary Material

Supplementary Information

## Figures and Tables

**Table 1:** Surface areas and pore volumes of the selected MOGs.

**S. No**	**Synthesized Gel**	**Surface Area [m**^**2**^**/g]**	**Pore Volume (cm**^**3**^**/g)**
1	MOA-1	751	2.601
2	MOA-2	829	3.482
3	MOA-3	1861	9.737
4	MOA-4	1016	5.855
5	MOA-5	1747	5.685
6	MOX-2	637	0.361
7	MOX-3	1289	0.88
8	MOX-4	428	0.282

**Table 2:** Summary of Gels with respect to surface areas and pore volumes.

**S. No.**	**Gel Name**	**Type of Gel**	**Surface Area (m**^**2**^**/g)**	**Pore Volume (cm**^3^**/g)**	**References**
1	Polyurethane	Aerogel	300	0.24	25
2	Tetra ethoxysilane	Aerogel	1108	4.7	2
3	Fe-BTC	Aerogel	1618	5.6	7
4	Fe-BDC	Aerogel	1016	0.67	28
5	Al-BDC	Aerogel	1560	5.41	15
6	Al-BTC	Aerogel	1638	6.26	16
7	Al-BTC	Aerogel	1266	7.15	16
8	Al-BTC	Aerogel	1795	4.5	30
9	0.5Fe-0.5Al-BTC	Aerogel	1861	9.737	This work
10	0.5Fe-0.5Al-BTC	Xerogel	1289	0.88	This work

**Table 3:** Kinetic parameters for adsorption of MO on MOA-3.

**C**_**o**_ **(mg L**^**−1**^)	**q**_**e**_ **(exp) (mg g**^**−1**^)	**Pseudo-second order kinetic model**		
		**q**_**e**_**(cal) (mg g**^**−1**^)	**K**_**2**_ **(g mg**^**−1**^ **min**^**−1**^)	**R**^**2**^
50	45.9	46	0.03	0.9994
100	93.7	93	0.008	0.99998
200	179	179	0.004	0.9999
