# Supplementary material for: Hierarchical Heteroaggregation of Binary Metal-Organic Gels with Tunable Porosity and Mixed Valence Metal Sites for Removal of Dyes in Water
Source: Sci Rep. 2015 May 27;5:10556. doi: 10.1038/srep10556 (PMC4444977; doi:10.1038/srep10556)
Supplement: Supplementary Information [file srep10556-s1.pdf]

Supporting Information

Hierarchical Heteroaggregation of Binary Metal-Organic Gels with Tunable Porosity and Mixed Valence Metal Sites for Removal of Dyes in Water

*Asif Mahmood, Wei Xia, Nasir Mahmood, Qingfei Wang, Ruqiang Zou**

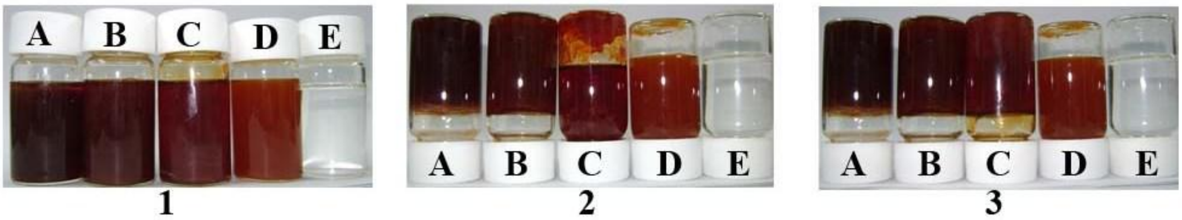

Figure S1: Growth of MOGs; (A) MOA-1, (B) MOA-2, (C) MOA-3, (D) MOA-4 and (E) MOA-5

The growth of MOGs at different times intervals after mixing the metal and organic ligand solutions at room temperature is shown in this picture. Figure S1-1 represent mixture formed just after mixing the starting species, Figure 2 represent mixtures after 20 minutes while Figure 3 represent the mixtures after 1 hour. It is quite clear that MOA-4 and MOA-5 do not form any solid gel at room temperature.

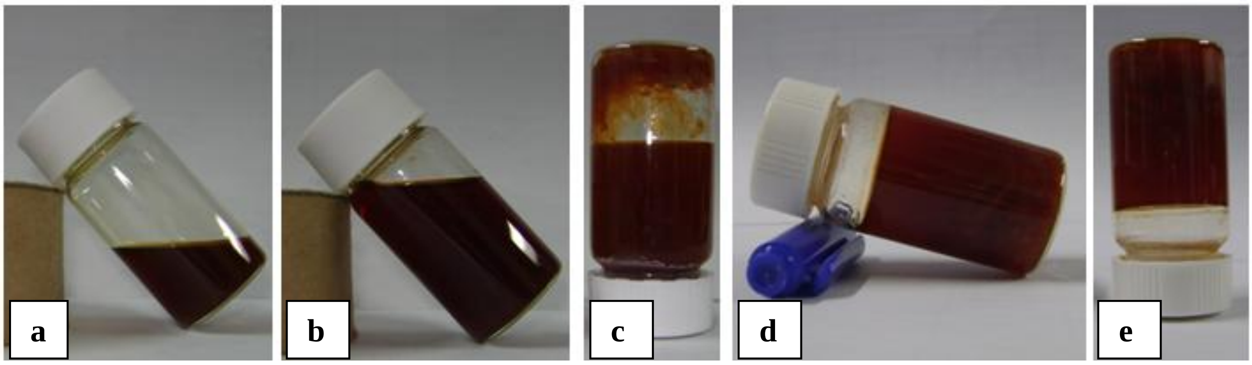

Figure S2: Growth of MOA-3. These pictures show formation of solid gel from the initial mixture of the mixed metals and organic ligand. Figure S2-a represent the metal solution and S2-b represent mixture of metal and ligand solutions at room temperature. It is clear from Figure S2-c that incomplete gel is formed in case of mixed metal MOGs and then formation of solid gel (Figure S2-d, S2-e) on standing for further time

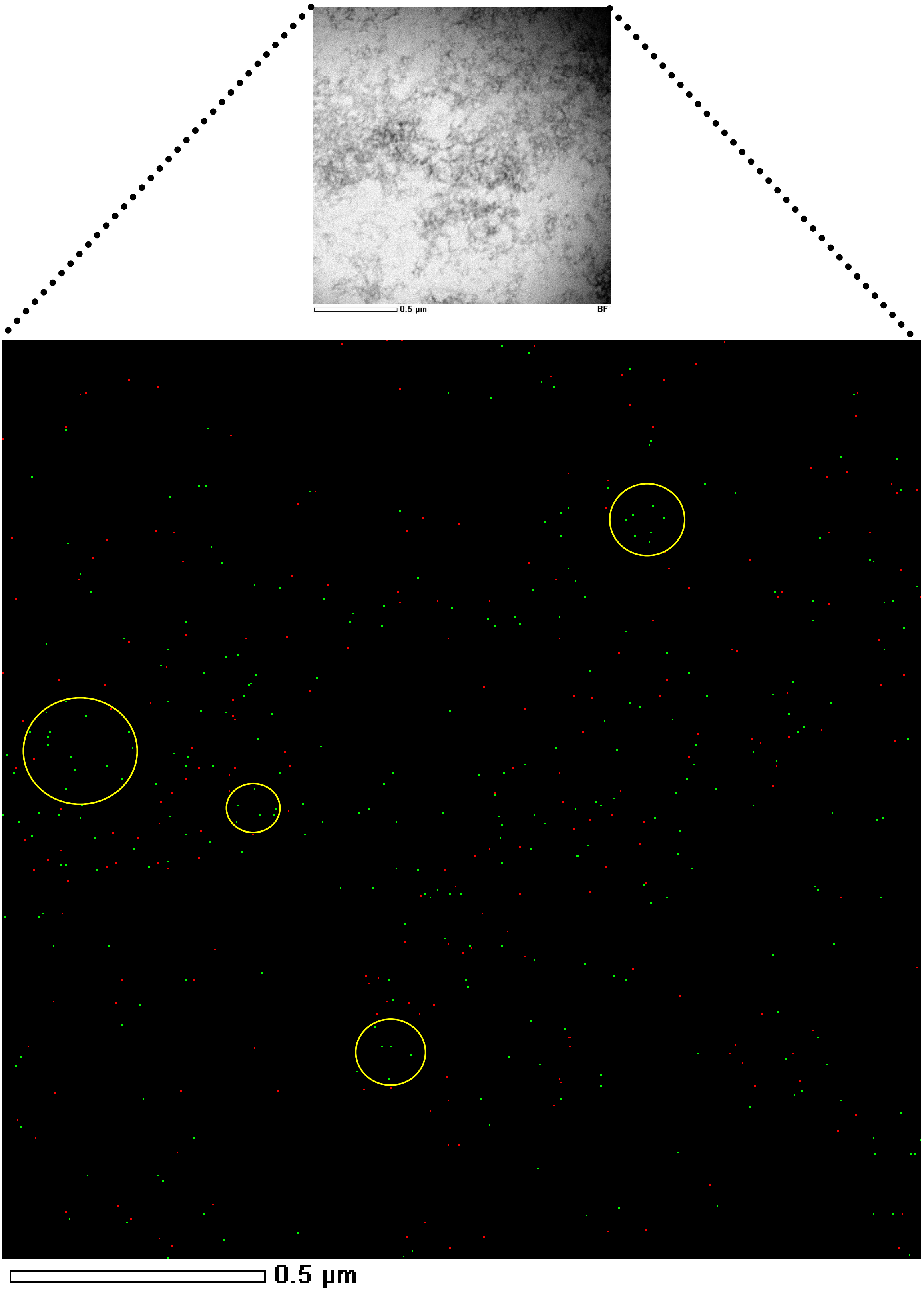

Figure S3. TEM image and elemental mapping. Representing the distribution of the elements in the MOA-3. Fe (green), Al (red)

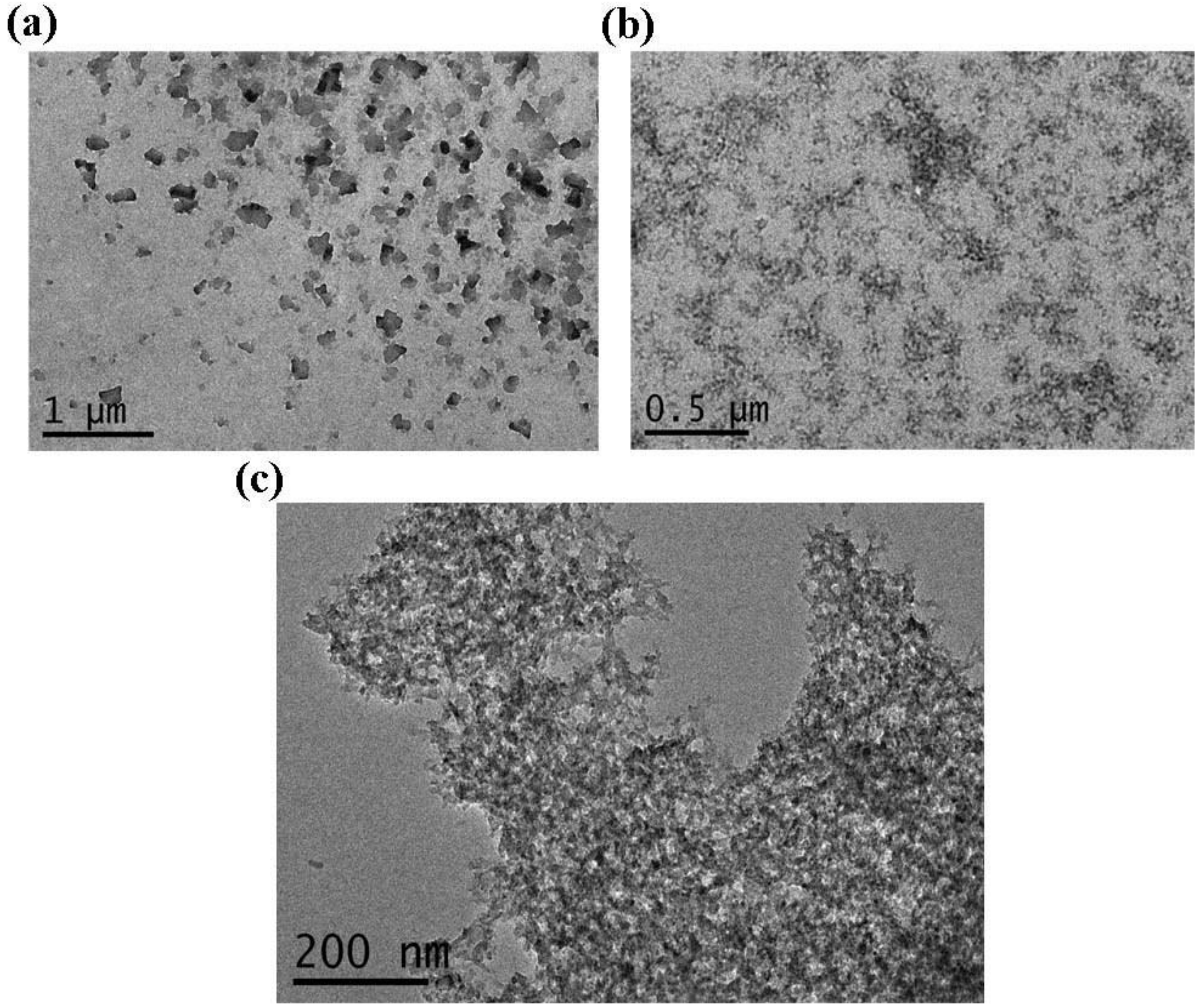

Figure S4: TEM analysis of MOA-3 synthesized at (a) room temperature, (b) 80 °C, (c) 120 °C

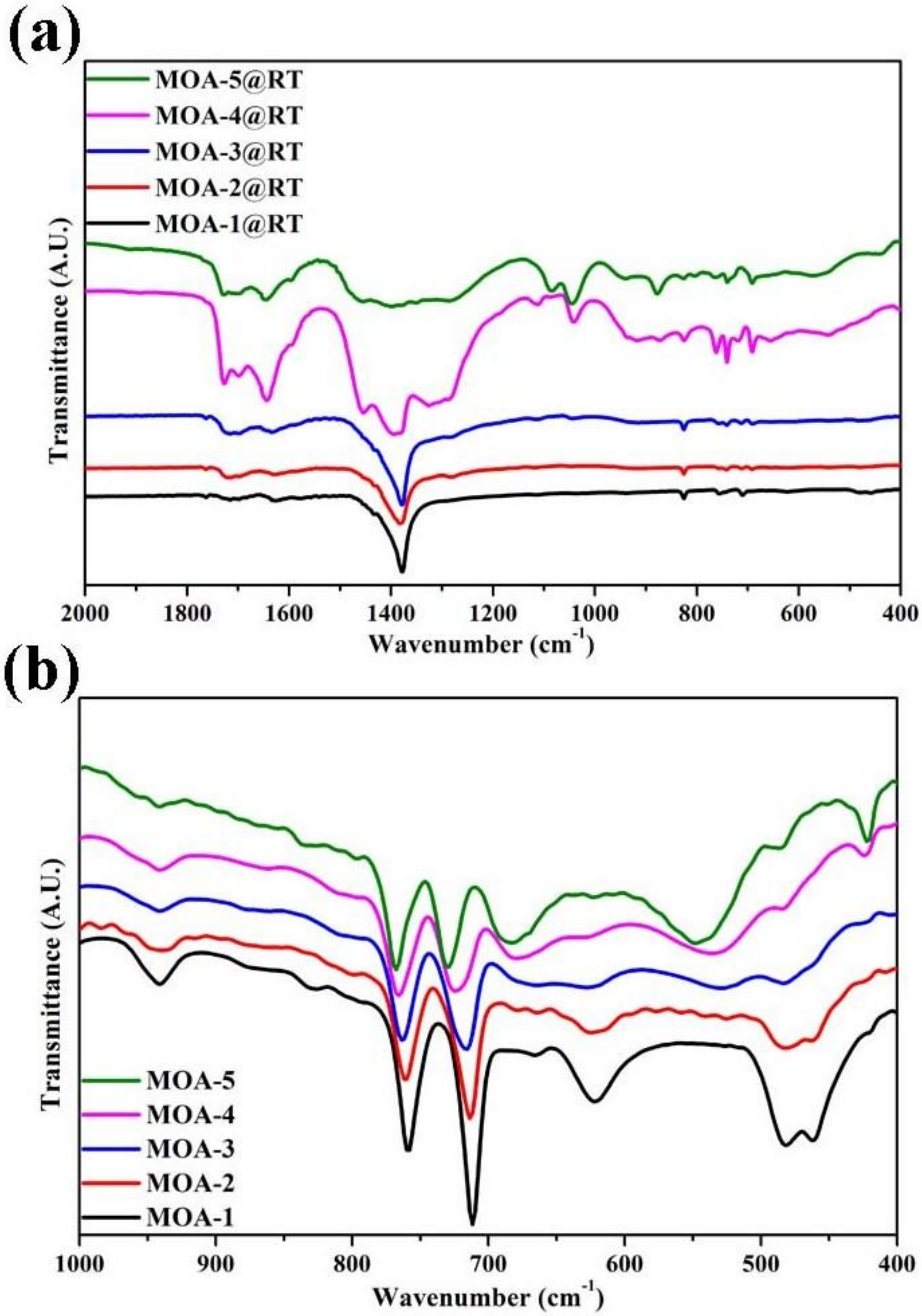

Figure S5: FTIR spectra of MOGs synthesized at (a) room temperature and at (b) 120 °C

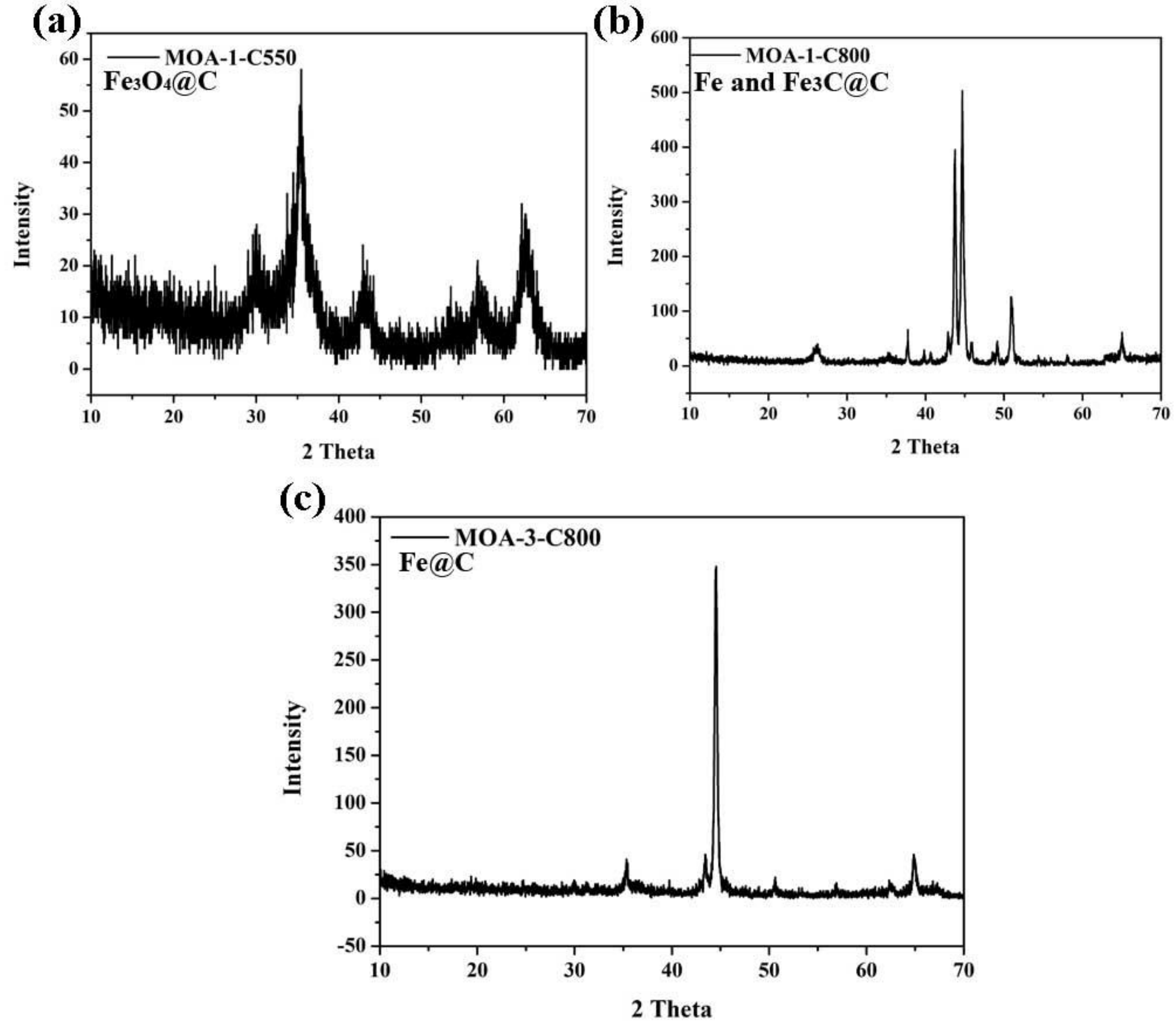

Figure S6: XRD analysis of residue at different heating temperatures

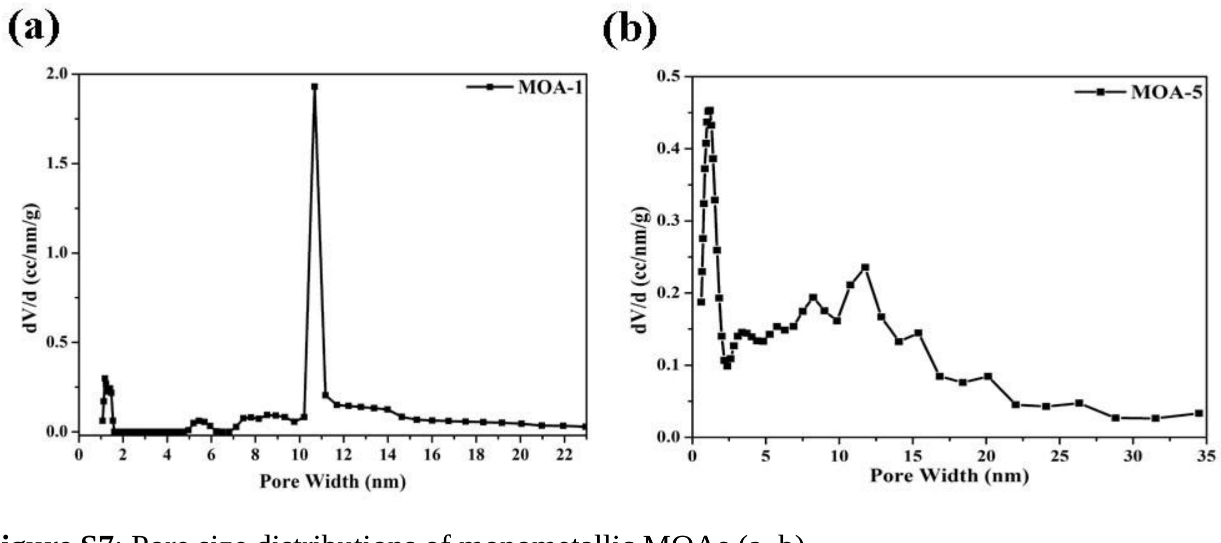

Figure S7: Pore size distributions of monometallic MOAs (a, b).

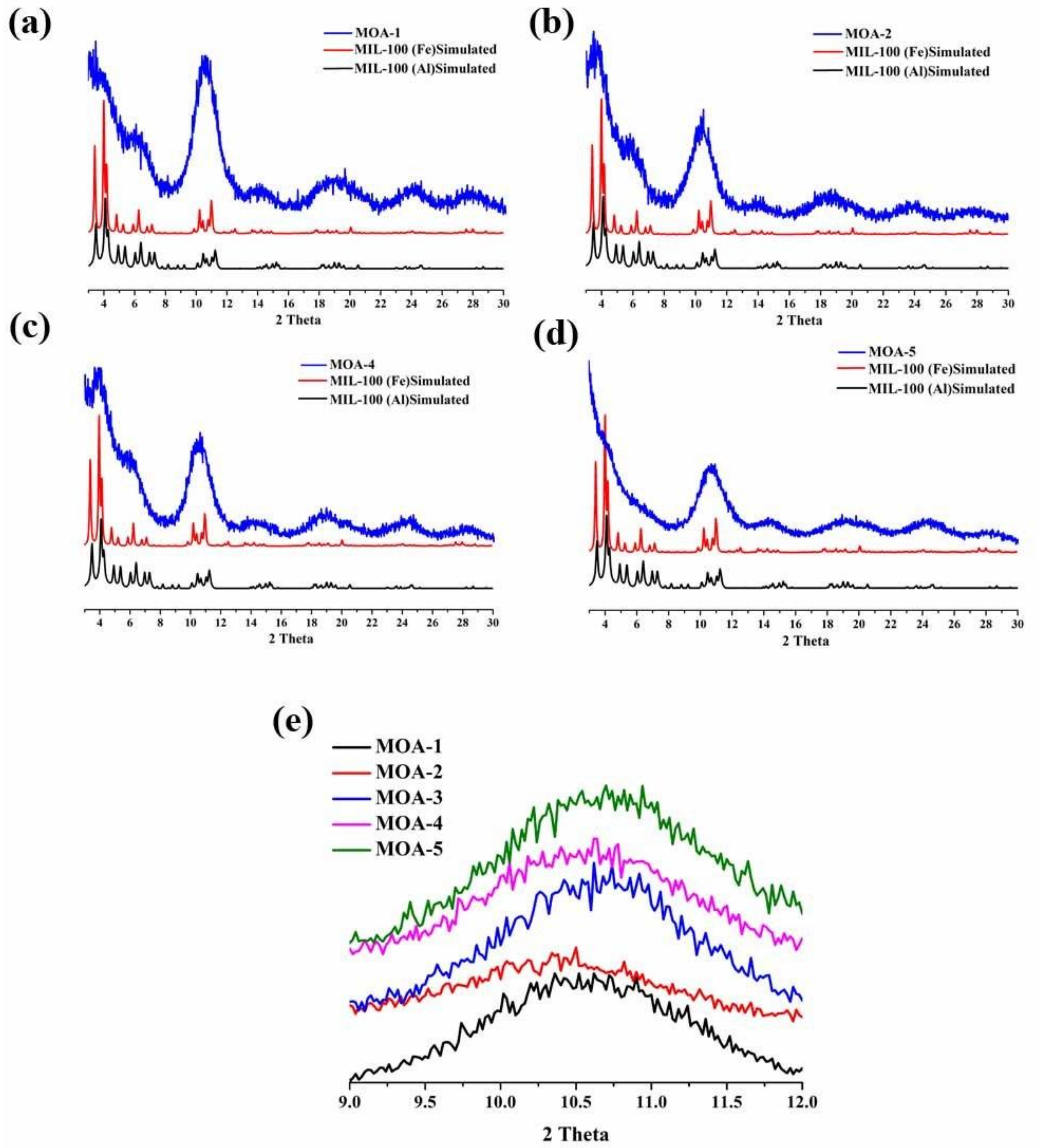

Figure S8: XRD analysis of MOGs, Pattern of (a) MOA-1, (b) MOA-2, (c) MOA-4, (d) MOA-5 with the simulated patterns of MIL-100 (Fe)[1] and MIL-100 (Al)[2]. Figure (e) represents peak comparison of aerogels.

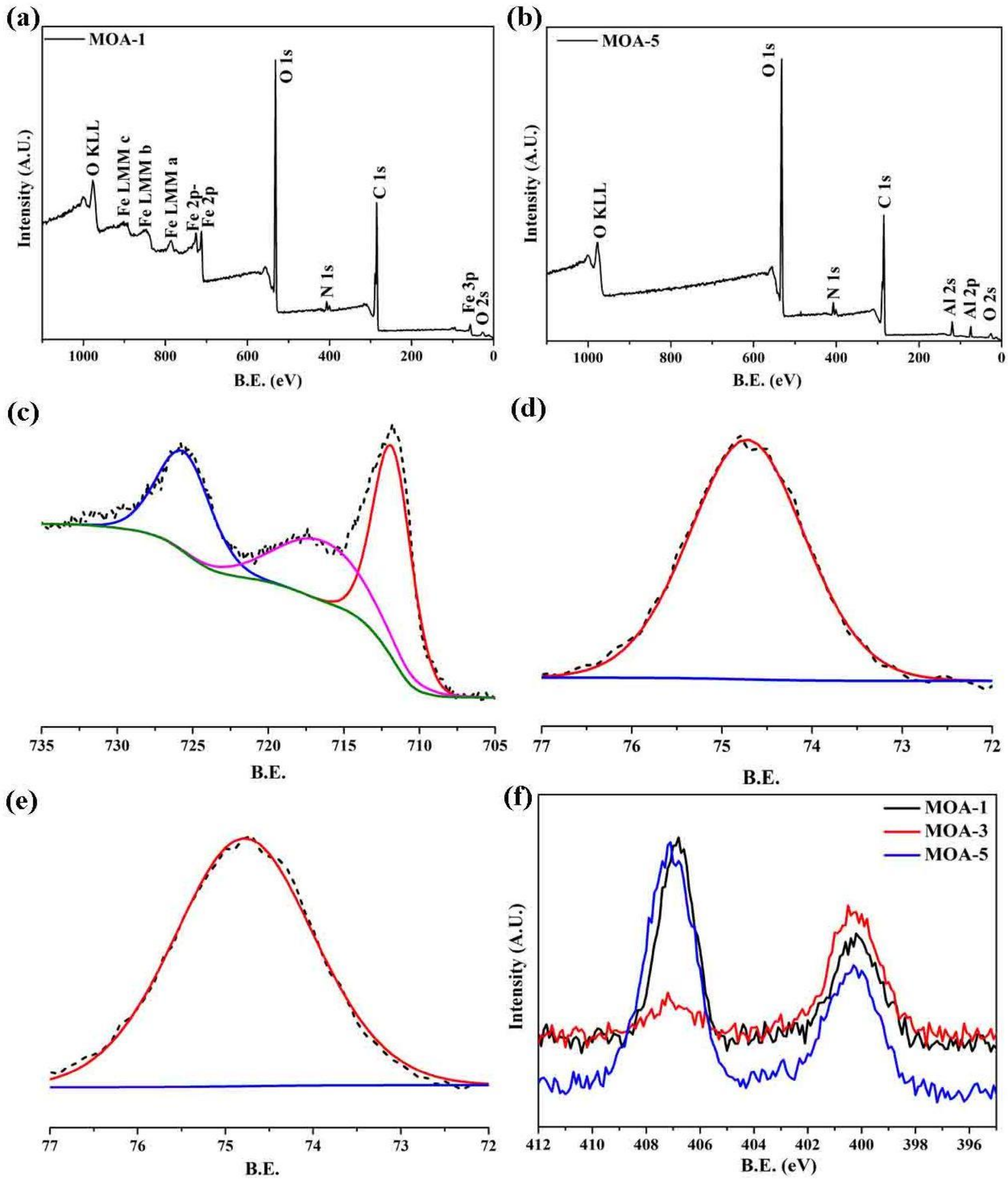

Figure S9: (a, b) XPS spectra of MOA-1 and MOA-5 respectively, (c) Fitted XPS spectrum of Fe from MOA-1, (d) Fitted XPS spectrum of Al from MOA-3 (e) Fitted XPS spectrum of Al from MOA-5, (f) Comparison of nitrogen concentration in MOGs.

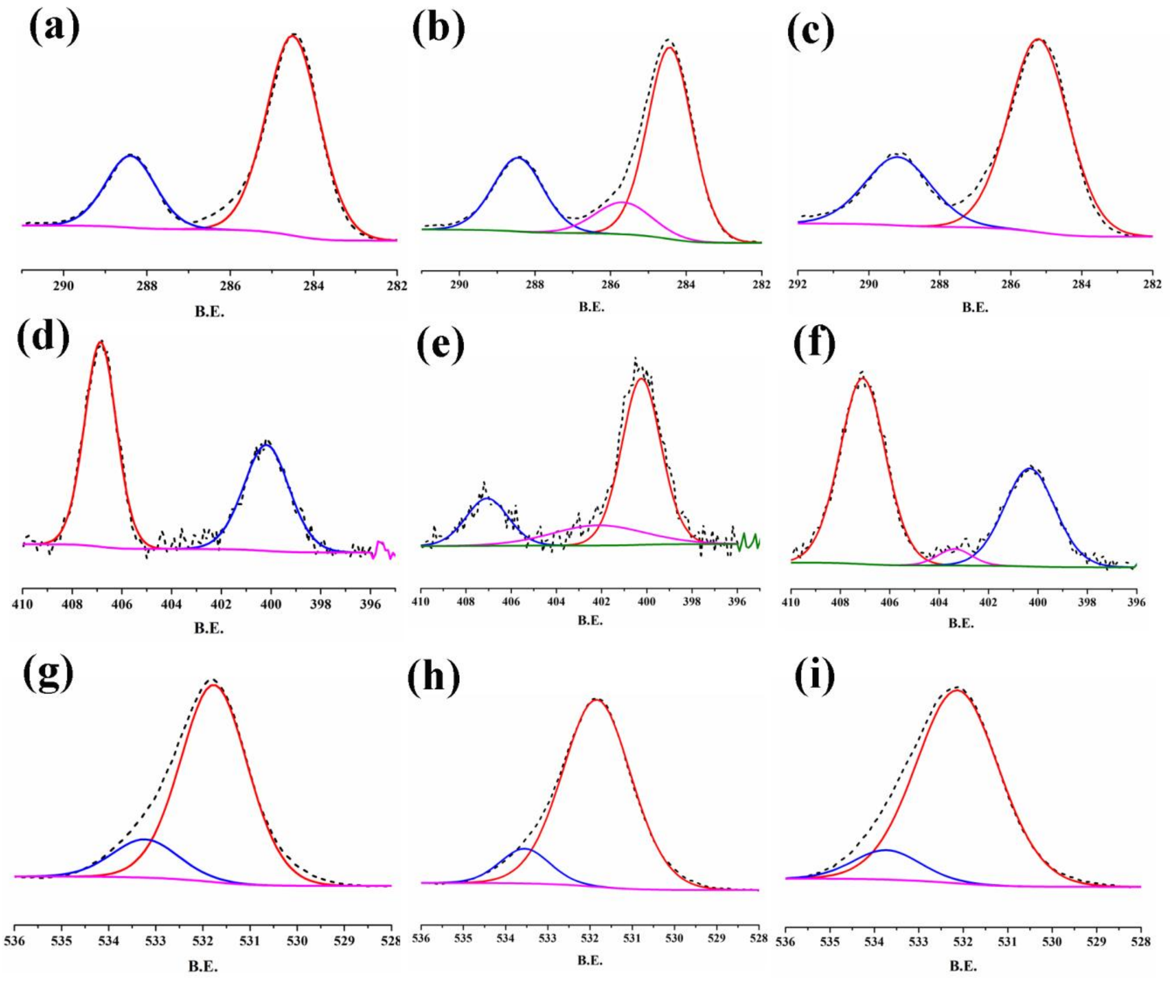

Figure S10: Deconvoluted XPS signals for Carbon, Nitrogen and Oxygen from MOGs. Figure (a, d, g) represents deconvoluted signals for Carbon, Nitrogen and Oxygen respectively from MOA-1, (b, f, h) represents deconvoluted signals from Carbon, Nitrogen, Oxygen respectively from MOA-3 and (c, f, i) represents signals for Carbon, Nitrogen and Oxygen respectively from MOA-5.

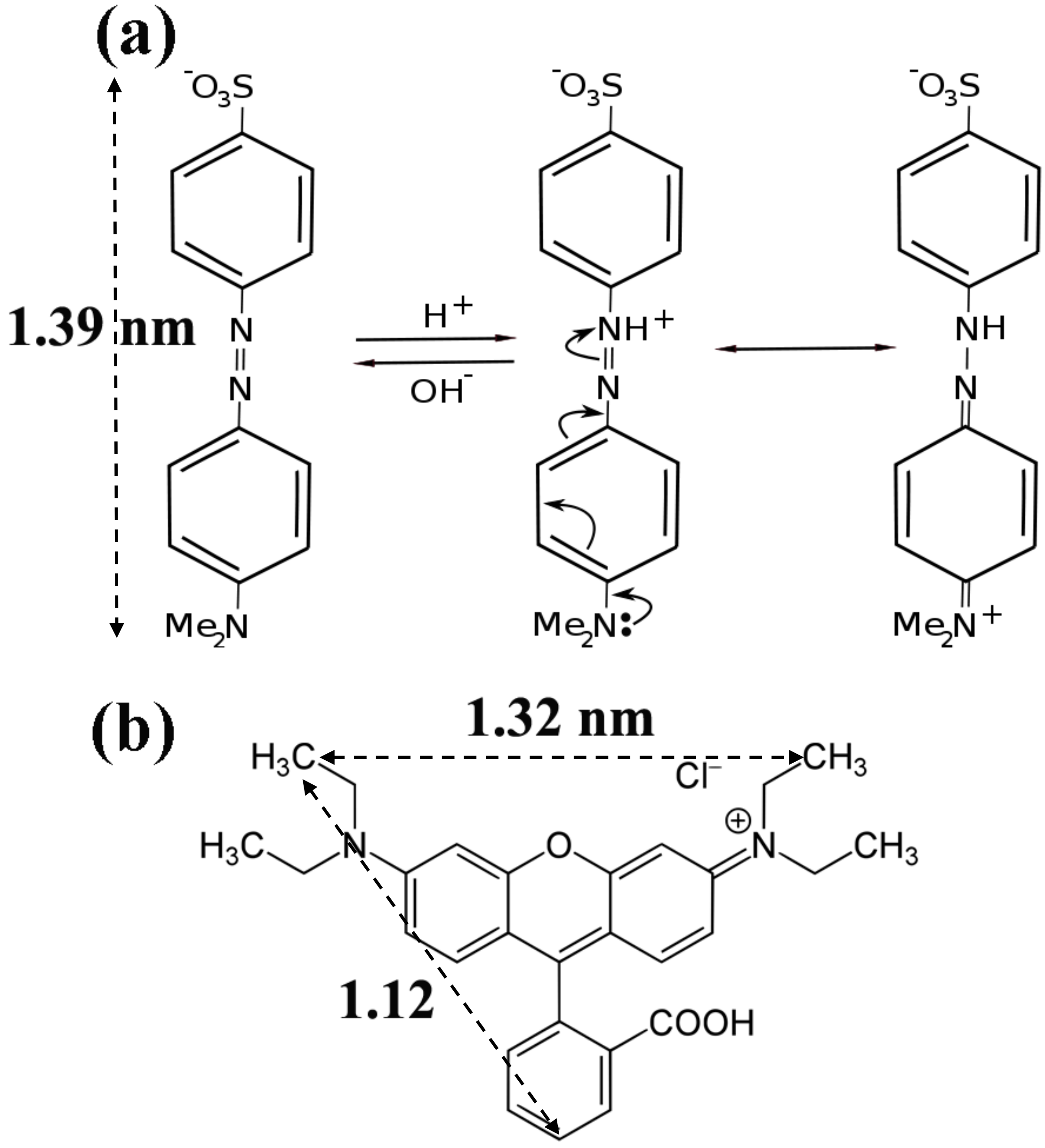

Figure S11: Chemical structures of used dye molecules. Ionic state of Methyl orange molecules and their diameters are drawn in Figure (a). Rhodamine B molecule is shown in Figure (b).

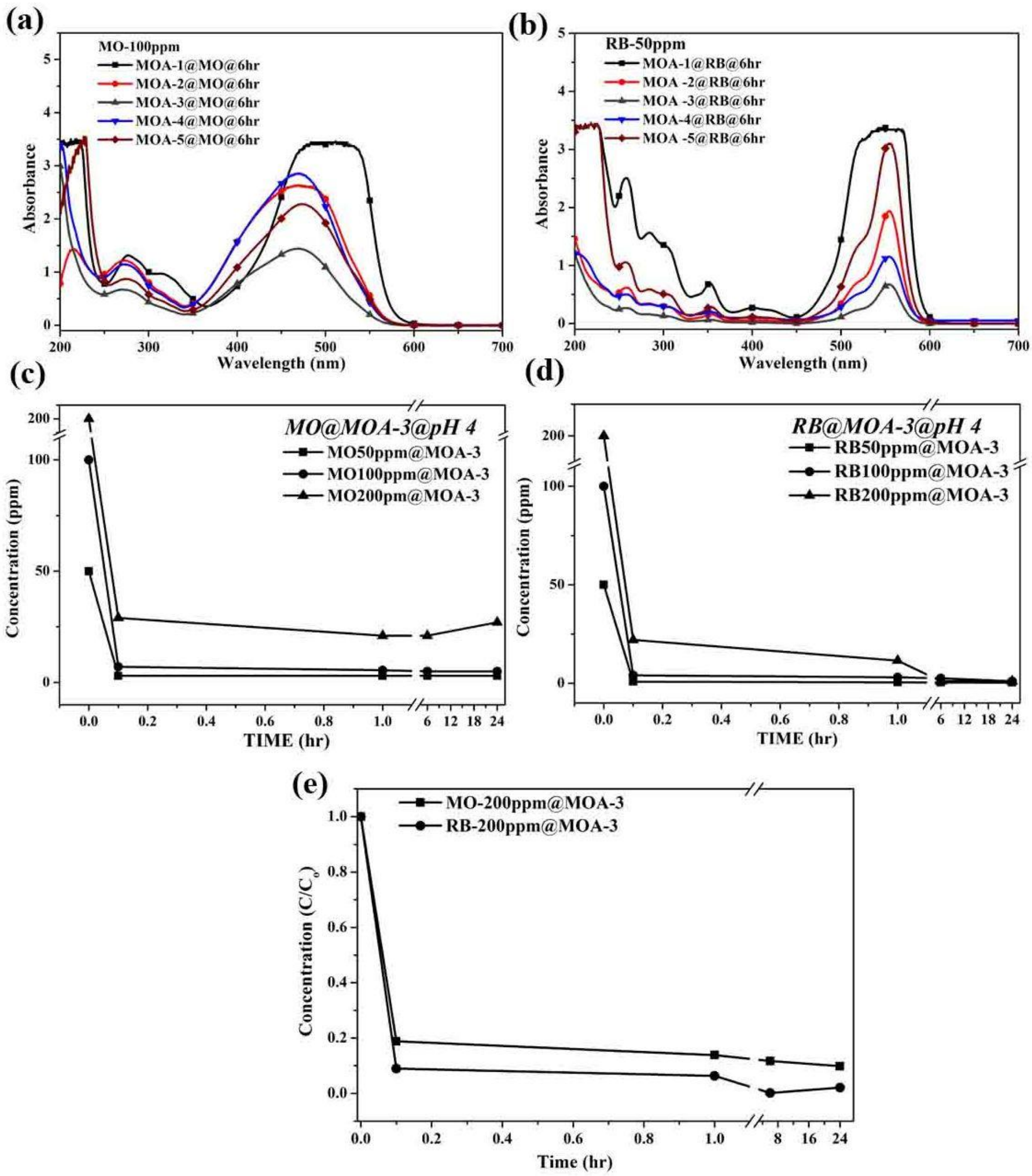

Figure S12: a-b represents the adsorption of dyes (MO and RB) on MOGs at pH~7 after 6 hours exposure time. Figure S12 (c-e) represent the relation of dye concentration vs time on adsorbent (MOA-3).

Desorption Experiments

Several solvents were tried to evaluate the desorption study of dye molecules from MOGs. 10% Acetic acid solution in ethanol provided ideal results for desorption of dyes. The core structure of MOGs was retained as demonstrated by XRD analysis shown below.

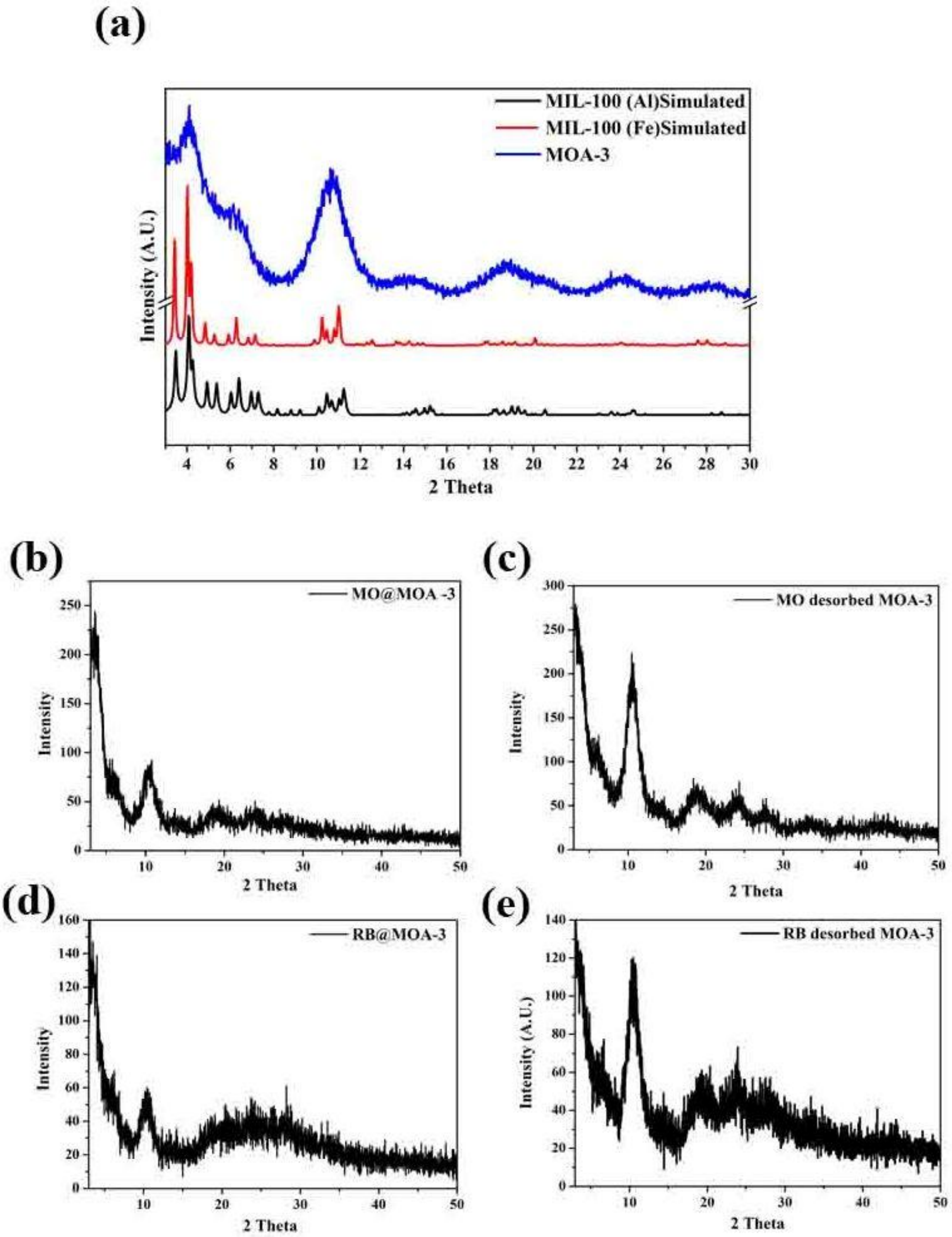

Figure S13: XRD analysis of (a) as synthesized MOA-3, (b) MO@MOA-3, (c) MO-desorbed from MOA-3, (d) RB@MOA-3 and (e) RB desorbed from MOA-3.

References

1. Horcajada, P., Surble, S., Serre, C., Hong, D. Y., Seo, Y. K., Chang, J. S., Greneche, J. M., Margiolaki, I. & Ferey, G. Synthesis and Catalytic Properties of MIL-100(Fe), An Iron(III) Carboxylate With Large Pores. *Chem. Commun.*, 2820-2822 (2007).
2. Volkringer, C., Popov, D., Loiseau, T., Ferey, G. r., Burghammer, M., Riekel, C., Haouas, M. & Taulelle, F. Synthesis, Single-Crystal X-ray Microdiffraction, and NMR Characterizations of the Giant Pore Metal-Organic Framework Aluminum Trimesate MIL-100. *Chem. Mat.* **21**, 5695-5697 (2009).
